# Immune Regulatory Cells in Inflammation, Infection, Tumor, Metabolism, and Other Diseases

**DOI:** 10.1155/2018/4170780

**Published:** 2018-06-12

**Authors:** Qingdong Guan, Cheng Xiao, Minggang Zhang

**Affiliations:** ^1^Cellular Therapy Laboratory, CancerCare Manitoba, Winnipeg, MB, Canada; ^2^The Institute of Clinical Research and Translational Medicine, Gansu Provincial Hospital, Lanzhou, China; ^3^Institute of Clinical Medicine, China-Japan Friendship Hospital, Beijing, China; ^4^Immunology Program, Memorial Sloan-Kettering Cancer Center, New York, NY, USA

The immune system is the host defense against infection, inflammation, tumor, and other diseases. The regulatory arm of immune system, including regulatory immune cells, regulatory complement, and regulatory cytokines, plays important roles in controlling immune responses and shutting down inflammation. However, cancer and some pathogens may escape and/or limit immune responses by driving immune regulatory cells. The balance of regulatory arm and effector arm of immune system maintains immune homeostasis. The recent advance has greatly increased our understanding of immune regulatory cells in health and pathogenesis of inflammation, infection, tumor, and other diseases. Immune regulatory cells are a large group of cells, such as regulatory T cells, myeloid-derived suppressor cells, mesenchymal stromal/stem cells, regulatory dendritic cells, and regulatory eosinophils. One of the keys to control infection, inflammation, tumor, and other diseases is to improve the understanding of immune regulatory cells in these situations.

In this special issue, we present original research articles as well as review papers on the role of immune regulatory cells with other immune cells in inflammation, infection, tumor, and other diseases. *Leishmania (L.) infantum chagasi* causes both visceral leishmaniasis and nonulcerated or atypical cutaneous leishmaniasis (NUCL). NUCL is characterized by mononuclear inflammatory infiltration of the dermis. By far, little is known about the pathogenesis of NUCL. G. V. A. Flores et al. evaluated the regulatory response in situ in skin lesions of patients affected by NUCL. By immunohistochemistry, CD4^+^, FoxP3^+^, TGF-*β*^+^, and IL-10^+^ cells were found in the dermis with inflammatory infiltration in all studied cases and at higher densities compared to the normal skin controls. A strong correlation was observed between CD4^+^ and FoxP3^+^ cells, and a moderate correlation was observed between FoxP3^+^ and TGF-*β*^+^, suggesting that T regulatory FoxP3^+^ cells and the regulatory cytokines, especially TGF-*β*, play an important role in the immunopathogenesis of NUCL, modulating a cellular immune response in the skin, avoiding tissue damage, and leading to low tissue parasitic persistence.

In recent years, studies have generated an accumulating wealth of evidence on the role of IL-17-producing cells in protective immunity to intracellular bacterial pathogens, such as *Mycobacterium tuberculosis* and *Chlamydia trachomatis*, which are one of the most important types of pathogens that inflict significant socioeconomic burden across the globe. Y. Li et al. summarized the recent progress on the functions and mechanisms by which Th17/IL-17 responds to intracellular bacterial infections. L. Sun et al. showed that V*γ*1^+^ T and V*γ*4^+^ T cells were the major proliferative cell subsets of *γδ* T cell during *Chlamydia muridarum* lung infection in mice; moreover, V*γ*4^+^ T cells were the major IL-17 and IFN*γ*-producing *γδ* T cell subsets at the early period of *Chlamydia muridarum* lung infection. These findings provide new insights into the mechanisms bridging innate and adaptive immunity during lung chlamydial infections.

Recent advances have demonstrated that microbiota play important roles in many kinds of diseases. Q. Li et al. summarized how changes of the gut microbiota affect the physiological and pathological properties of the intestinal immunomodulatory cells, thus regulating diabetes mellitus. Understanding this bridge role of intestinal immunomodulatory cells may clarify the mechanisms by which the gut microbiota contributes to diabetes mellitus. Periodontitis is a chronic immunoinflammatory disease, in which the disruption of the balance between host and microbiota interactions is the key to the onset and progression of the disease. The immune homeostasis associated with periodontal health requires a regulated immunoinflammatory response, during which the presence of regulatory T cells (Treg) is essential to ensure a controlled response that minimizes collateral tissue damage. C. Alvarez et al. presented a comprehensive summary of regulatory T lymphocytes in periodontitis from bench to bedside.

Understanding the phenotypic and functional properties of tumor infiltrating Treg is essential to effectively and specifically target these cells in cancer therapy without compromising immune homeostasis in general. G. R. Lee summarized recent advances relating to tumor-infiltrating Treg in the tumor microenvironment, with particular emphasis on their accumulation, phenotypic, and functional properties, and targeting to enhance the efficacy of antitumor treatment.

Accumulating evidence suggests that neuroinflammation plays a critical role in postoperative cognitive dysfunction (POCD). Y. Liu and Y. Yin summarized the roles of immune regulatory cells with other immune cells and crosstalk between them in the pathogenesis of POCD, which may uncover promising therapeutic targets for POCD treatment and prevention.

Monocytes play an essential role in the pathogenesis of acute exacerbation of chronic obstructive pulmonary disease (AECOPD). J. Yang et al. analyzed monocyte subpopulation in the peripheral blood of healthy volunteers and AECOPD patients at the stages of admission and remission after clinical therapy, and they found a dramatic increase of a previously unreported population of large circulating atypical monocytes in AECOPD patients, characterized by higher forward scatter and lower side scatter, higher expression of CD16, intercellular adhesion molecule 1 (ICAM-1) and chemotactic protein-1 receptor-2 (CCR2), and lower expression of MHC class II antigen (HLA-DR) than typical monocytes. Further, it was found that the percentage of circulating atypical monocytes in total monocytes correlated with the length of hospital stay and disease duration. The circulating atypical monocytes might have the potential to serve as a biomarker in the diagnosis and prognosis of AECOPD.

Y. Wang et al. showed that the expression of mitochondrial protein nicotinamide adenine dinucleotide dehydrogenase (ubiquinone) Fe-S protein 3 (NDUFS3) was significantly decreased in the sperm of patients with Asthenozoospermia (AS), and DJ-1 may play a role in maintaining mitochondrial function by means of the association with NDUFS3 during spermatogenesis in the testes. The interaction between DJ-1 and NDUFS3 in rat testes was weakened by ornidazole treatment. It showed that downregulation of DJ-1 and NDUFS3 expression likely contributes to mitochondrial dysfunction, which may underlie AS pathogenesis.

Overall, we believe that these articles may contribute to improve our knowledge of immune regulatory cell and other immune cell-mediated immune mechanisms in infection, inflammation, tumor, and other diseases, to provide insights into designing of effective immunodiagnostic tools, and to present rational basis for the development of potential therapeutic strategies.

